# Predator cannibalism can shift prey community composition toward dominance by small prey species

**DOI:** 10.1002/ece3.8894

**Published:** 2022-05-07

**Authors:** Kunio Takatsu

**Affiliations:** ^1^ 28499 Graduate School of Environmental Science Hokkaido University Horonobe Japan; ^2^ Department of Fish Ecology and Evolution Centre for Ecology, Evolution & Biogeochemistry Eawag: Swiss Federal Institute of Aquatic Science and Technology Kastanienbaum Switzerland

**Keywords:** cannibalism, gigantism, *Hynobius retardatus*, predator–prey interaction, top‐down effects

## Abstract

Cannibalism among predators is a key intraspecific interaction affecting their density and foraging behavior, eventually modifying the strength of predation on heterospecific prey. Interestingly, previous studies showed that cannibalism among predators can increase or reduce predation on heterospecific prey; however, we know less about the factors that lead to these outcomes. Using a simple pond community consisting of *Hynobius retardatus* salamander larvae and their associated prey, I report empirical evidence that cannibalism among predators can increase predation on large heterospecific prey but reduce that on small heterospecific prey. In a field‐enclosure experiment in which I manipulated the occurrence of salamander cannibalism, I found that salamander cannibalism increased predation on frog tadpoles but reduced that on aquatic insects simultaneously. The contrasting effects are most likely to be explained by prey body size. In the study system, frog tadpoles were too large for non‐cannibal salamanders to consume, while aquatic insects were within the non‐cannibals’ consumable prey size range. However, when cannibalism occurred, a few individuals that succeeded in cannibalizing reached large enough size to consume frog tadpoles. Consequently, although cannibalism among salamanders reduced their density, salamander cannibalism increased predation on large prey frog tadpoles. Meanwhile, salamander cannibalism reduced predation on small prey aquatic insects probably because of a density reduction of non‐cannibals primarily consuming aquatic insects. Body size is often correlated with various ecological traits, for instance, diet width, consumption, and excretion rates, and is thus considered a good indicator of species’ effects on ecosystem function. All this considered, cannibalism among predators could eventually affect ecosystem function by shifting the size composition of the prey community.

## Graphical abstract

Cannibalism among predators can increase or reduce predation on heterospecific prey. This study provides empirical evidence that the direction of predator cannibalism effects can differ depending on prey body size using a simple pond system consisting of predatory salamander larvae and their associated prey community composed of frog tadpoles and aquatic insects.

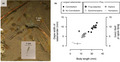

## 1 INTRODUCTION

Predators can strongly influence the abundance of their heterospecific prey, eventually affecting species richness and composition of prey community and its ecosystem functions. Population size (i.e., density) and foraging behavior of predators are fundamental elements in determining the predatory effects on their heterospecific prey (Ohgushi, 2012; Werner & Peacor, 2003; Wootton, 1994). Notably, because individuals constituting a focal predator population can differ in their foraging behavior (Bolnick et al., 2011; Miller & Rudolf, 2011; Violle et al., 2012), the net effect of the predator population on their prey community would be the sum of each individual's effects. Therefore, studying factors affecting predator density and individual foraging behavior and how the changes in predator density and behavior are translated into the predatory effects will provide knowledge to better understand the spatial and temporal variation in community composition (Ohgushi, 2012; Werner & Peacor, 2003; Wootton, 1994).

Particularly for predators, cannibalism is a key intraspecific interaction affecting population density but also differentiating foraging behaviors of individuals constituting the population (Claessen et al., 2004; Fox, 1975; Polis, 1981). However, predicting how cannibalism among predators modifies their effects on heterospecific prey is challenging because cannibalism‐induced changes in density and foraging behavior of predators can have contrasting impacts on the strength of predatory effects. In general, cannibalism among predators causes the following changes in their population. Firstly, cannibalism among predators reduces their density (Fox, 1975). Secondly, when cannibalism occurs, potential victims (i.e., non‐cannibals) reduce their foraging activity to reduce their likelihood of being detected by the cannibals (Rudolf, 2012; Sih, 1982; Wissinger et al., 2010). Moreover, especially in predators in aquatic ecosystems such as fish, amphibian larvae, and aquatic insects, cannibalism often causes enhancement of growth of individuals that succeeded in consuming conspecifics (i.e., cannibals; Hardie & Hutchings, 2014; Kishida, 2011; Sniegula et al., 2019). The reductions in both density and foraging activity of non‐cannibals are likely to reduce predation on heterospecific prey. In contrast, the enhancement of the growth can result in the improvement of the foraging ability of cannibals, thereby increasing predation on heterospecific prey. Notably, reductions in non‐cannibals’ density and foraging activity, and enhancement of cannibals’ growth occur together within a predator population due to cannibalism. Therefore, the net effects of predator cannibalism on the strength of predation on heterospecific should differ depending on the relative importance between reductions in non‐cannibals’ density and foraging activity and improvement of cannibals’ foraging ability. For example, cannibalism among predators should reduce predation on their heterospecific prey if the reductions in non‐cannibals’ density and foraging activity are more significant than cannibals’ improved foraging ability in determining the strength of predation. Indeed, previous studies examining how cannibalism among predators affects their heterospecific prey showed both reductions (Crumrine, 2010a; Persson et al., 2003; Rudolf, 2006, 2012) and intensifications (Takatsu & Kishida, 2015, 2020) of the strength of predation on the prey as a result of predator cannibalism. The differences in the reported patterns among studies raise a question; what factors affect the relative importance between the reductions in non‐cannibals’ density and foraging activity and the improvement of cannibals’ foraging ability in determining the strength of predation on heterospecific prey?

The body size of heterospecific prey can play a pivotal role in determining the relative importance between the reductions in non‐cannibals’ density and foraging activity and the improvement of cannibals’ foraging ability for the following reasons. Firstly, predators often co‐occur with different‐sized prey species, and also the prey community size composition varies across space and time. Secondly, particularly for carnivorous fish and amphibian larvae, which are often top predators in freshwater food webs and exhibit cannibalism (Fox, 1975), the upper limit of consumable prey size is often restricted by the predator's gape size (Montori et al., 2006; Nosaka et al., 2015). It is worth mentioning that the upper limit of consumable prey size increases with the growth of predator individuals (Werner & Gilliam, 1984). All this considered, the direction of the net effect of predator cannibalism on the strength of predation on heterospecific prey can differ depending on whether focal prey are larger or smaller than the upper limit of consumable prey size for non‐cannibals.

For example, when focal prey are larger than or similar to the upper limit of consumable prey size for non‐cannibals, non‐cannibals rarely consume the prey. Thus, the strength of predation on the large prey is less likely to be affected by the reductions in non‐cannibals’ density and foraging activity. Meanwhile, if cannibals reach large enough size to consume the large prey easily, the strength of predation on the large prey should be affected by the growth enhancement of cannibals. If this is the case, the strength of predation on the large prey should be intensified by cannibalism among predators eventually. On the other hand, when focal prey are smaller than the upper limit of consumable prey size for non‐cannibals, both cannibals and non‐cannibals consume the prey. Thus, the strength of predation on the small prey is likely to be affected both by reductions in non‐cannibals’ density and foraging activity and also by the improvement of cannibals’ foraging ability. Notably, only a few individuals within a population can cannibalize and grow into gigantic cannibals (Huss et al., 2010; Kishida, 2011). All this considered, reductions in non‐cannibal's density and foraging activity are likely to be more significant than the improvement of cannibals’ foraging ability in determining the strength of predation on the small prey. Therefore, the strength of predation on the small prey should be reduced by cannibalism among predators eventually. Moreover, when the focal prey community includes both small and large prey, cannibalism among predators can further reduce the strength of predation on the small prey if cannibals shift their diet from small to large prey. In support of the hypothesized importance of prey body size in determining the direction of predator cannibalism effects, previous studies showing that cannibalism among predators reduces predation on prey used predator–prey systems in which focal prey items were smaller than the upper limit of consumable prey size for non‐cannibals (e.g., predatory fish and their prey zooplankton; Persson et al., 2003). Also, previous studies showing that cannibalism among predators increases predation on prey used a system in which the focal prey item was larger than or similar to the upper limit of consumable prey size for non‐cannibals (predatory salamander larvae and their prey frog tadpoles; Takatsu & Kishida, 2015, 2020). However, so far, no studies have directly tested the hypothesis that cannibalism among predators can reduce predation on small heterospecific prey but increase predation on large heterospecific prey.

**FIGURE 1:**
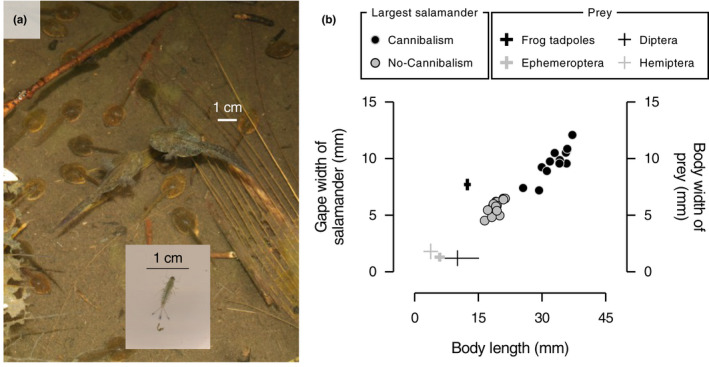
Size differences between salamander larvae, frog tadpoles, and aquatic insects. (a) Photo of salamander larvae, frog tadpoles, and Ephemeroptera larvae. (b) Relationship between body length and gape width of largest salamander in the enclosures on day 34 and relationships between body length and width of prey items. Filled circles represent the largest salamanders in the enclosures. The lines represent the standard deviation of body length and width of prey items

The simple pond community consisting of the *Hynobius retardatus* salamander larvae and their prey *Rana pirica* frog tadpoles and aquatic insects (e.g., mayfly and chironomid larvae and water boatman), which is commonly observed in small ponds (one to several tens m^2^) in Hokkaido, Japan, is an excellent model system to examine how cannibalism among predators affects the size composition of prey communities, because of the following knowledge. Firstly, salamander larvae consume their prey items, including conspecific victims (i.e., cannibalism), only when their gape size is larger than the body size of the prey (Nosaka et al., 2015). Secondly, there are significant differences in body size between *R*. *pirica* frog tadpoles and aquatic insects (Figure 1). In early spring, both *H*. *retardatus* salamanders and *R*. *pirica* frogs lay their eggs in the small ponds. Several weeks after the end of the reproductive season of the amphibians, the recruitment of larval aquatic insects occurs. Due to the differences in the reproductive timing as well as species‐specific body size, frog tadpoles, and salamander larvae are generally similar in their body size (i.e., frog tadpoles are large prey for the salamander larvae), while aquatic insects are far smaller than the larval amphibians (i.e., aquatic insects are small prey for the salamander larvae; Figure 1). For this study, I hypothesized that cannibalism among salamander larvae increases predation on frog tadpoles (large prey) but reduces predation on aquatic insects (small prey) at the same time. Here, I report results from a field‐enclosure experiment in which I manipulated the size structure of salamander larvae to control the occurrence of their cannibalism and then assessed how the effects of salamander cannibalism differ depending on differently sized prey items.

## 2 MATERIALS AND METHODS

### 2.1 Experimental environments

**FIGURE 2:**
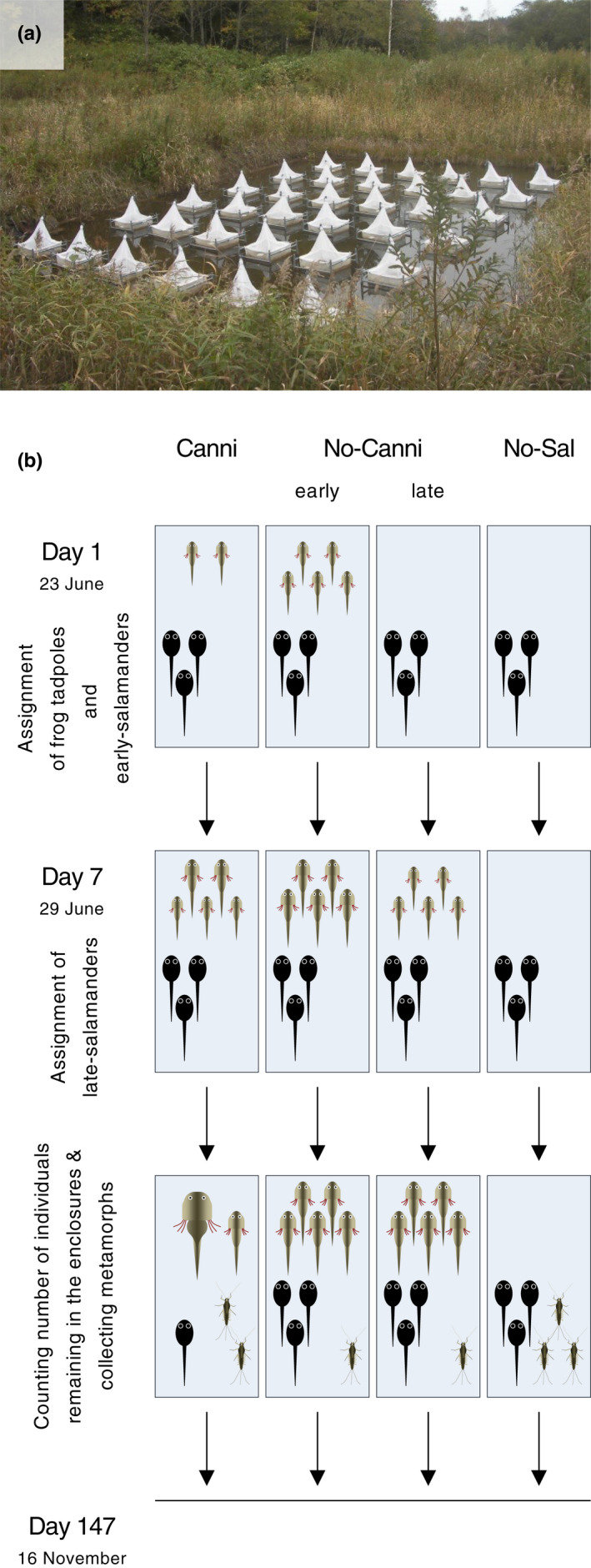
(a) Photograph of the 36 enclosures in the field pond in Teshio Experimental Forest of Hokkaido University. (b) Schematic diagram of the experiment. Canni, No‐canni, and No‐sal are abbreviations of Cannibalism, No‐cannibalism, and No‐salamander treatments, respectively

The experiment was conducted in a field pond (12 m × 15 m) in the Teshio Experimental Forest of Hokkaido University (45° 01′ 77.65″ N, 142° 01′ 47.71″ E) (Figure 2a). This pond has no canopy cover, and its bottom is composed of soil and small rocks at approximately 40 cm water depth. In this pond, I observed that *H*. *retardatus* and *R*. *pirica* eggs were laid from mid‐May until mid‐June and newly hatched amphibian larvae appeared in early June. Moreover, I observed the recruitment of several aquatic insects (mainly mayfly larvae, chironomid larvae, and water boatman) in the pond from mid‐July to the end of November.

I placed 36 cubic enclosures (60 cm × 60 cm × 60 cm) with polyvinyl chloride framing covered by 1 mm nylon mesh on all sides except the top side as an experimental unit in the pond on 21 June 2013. The intervals between enclosures were more than 50 cm (Figure 2a). An emergence trap was placed on the top side of each enclosure to prevent metamorphs of amphibians and emerging aquatic insects from escaping from the enclosures (Figure 2a). Periphyton grows on the mesh wall as resources for frog tadpoles and aquatic insects.

### 2.2 Experimental setting

A schematic diagram of the field experiment is shown in Figure 2b.

#### 2.2.1 Prey community

A prey community consisting of frog tadpoles and aquatic insects was established using the following methods. For frog tadpoles, I assigned 80 2‐week‐old laboratory‐reared frog tadpoles into each of the 36 enclosures on 23 June 2013 (i.e., day 1 of the experiment; Figure 2b). Mean ± *SD* body length (snout‐vent length) and body width of the frog tadpoles were 12.4 ± 0.8 mm and 7.7 ± 0.5 mm, respectively (*N* = 20) (Figure 1b). The density of the frog tadpoles (222 individuals m^2^) was within their natural range (Michimae, 2006). Methods for collecting and maintaining the frog tadpoles are described in Appendix A.

Since newly hatched aquatic insects can freely pass through the mesh wall of the enclosure when they are smaller than the mesh size (i.e., <1 mm), I used those which naturally colonized the enclosures for the experiment. While I observed several aquatic insects in the enclosures during the experiment, more than 97% of observed aquatic insects over the experimental period were composed of Ephemeroptera, Diptera, and Hemiptera (Appendix B). Hereafter, I focused on the Ephemeroptera, Diptera, and Hemiptera as focal aquatic insect prey in the experiment. Mean ± *SD* body length (from the tip of the head to the end of the abdomen) of Ephemeroptera larvae, Diptera larvae, and Hemiptera were 5.9 ± 1.2 mm (*N* = 20), 10.1 ± 5.1 mm (*N* = 20), and 3.8 ± 1.7 mm (*N* = 5), respectively (Figure 1b). Mean ± *SD* body width of Ephemeroptera larvae, Diptera larvae, and Hemiptera were 1.3 ± 0.2, 1.2 ± 0.7, and 1.8 ± 0.7 mm, respectively (Figure 1b). Therefore, those focal aquatic insects were far smaller than frog tadpoles (Figure 1b). These indicated that aquatic insects were small prey and the frog tadpoles were large prey for the salamander larvae. In addition to the aquatic insects, I also observed zooplankton, such as copepods, in the experimental pond. The recruitment of such zooplankton provided additional prey for the salamander larvae during the experimental period. However, I did not include zooplankton as focal prey in this study because zooplankton were smaller than the mesh size of the enclosures (1 mm) during their lifetime and were too difficult to count.

#### 2.2.2 Experimental treatments

To control cannibalism among salamander larvae, I followed the methods used in the previous studies (Takatsu & Kishida, 2015, 2020; Takatsu et al., 2017). Since the occurrence of cannibalism among salamander larvae depend greatly on size asymmetry between interacting individuals (Kishida et al., 2015), I manipulated size differences among salamander larvae (i.e., presence and absence of early‐ and late‐hatched salamander larvae) while keeping the total initial density of the salamander larvae constant across the treatments (i.e., 30 individuals, see below). In the experimental setting, the early‐hatched salamander larvae can grow until the late‐hatched salamander larvae hatch. This resulted in size asymmetry between them and eventually cannibalism (i.e., early‐hatched salamander larvae can cannibalize late‐hatched salamander larvae). I obtained the early‐ and late‐hatched salamander larvae by manually controlling the water temperature experienced by the embryos from a single egg cluster (i.e., half‐sib). Methodological details to obtain early‐ and late‐hatched salamander larvae are the same as those shown in Appendix A in Takatsu and Kishida (2015).

Using the 36 enclosures, I established the following four treatments: (1) Cannibalism, (2) No‐cannibalism‐early, (3) No‐cannibalism‐late, and (4) No‐salamander control treatments (Figure 2b). For the Cannibalism treatment, I assigned five early‐ and 25 late‐hatched salamander larvae into 14 enclosures. For No‐cannibalism‐early treatment, I assigned 30 early‐hatched salamander larvae into eight enclosures. For No‐cannibalism‐late treatment, I assigned 30 late‐hatched salamander larvae into eight enclosures. The difference between early‐(22 June) and late‐(28 June) hatched salamander larvae was 6 days. The salamander larvae were assigned to the relevant treatments 1 day after they hatched (i.e., days 1 and 7; Figure 2b). Mean ± *SD* body length and gape width of the salamander hatchlings at the assignment timing were 11.2 ± 0.8 and 2.8 ± 0.3 mm (*N* = 20), respectively. Methods for collecting and maintaining the salamander larvae are described in Appendix A. To make a No‐salamander control treatment, I did not assign any salamander larvae to the remaining six enclosures. I adopted the unbalanced replication design to avoid excessive use of the animals. This is because previous studies using the same larval amphibian system showed that variances of the demographic and trait level consequences were larger in the Cannibalism treatment than in the No‐cannibalism treatment (Takatsu & Kishida, 2015, 2020; Takatsu et al., 2017). Moreover, previous studies using the system showed that the mortality of the frog tadpoles in the absence of salamander larvae was very low (Kishida et al., 2014; Takatsu & Kishida, 2020). Each replicate was randomly assigned to one of the 36 field enclosures. The density of the salamander larvae (83 individuals m^2^) was within their natural range (Michimae, 2006).

### 2.3 Measurements of demographic variables

I counted the salamander larvae, frog tadpoles, and aquatic insects remaining in the enclosure on days 7, 24, 30, 34, 44, 57, 109, and 145. At the same time, I collected metamorphs of the amphibians and emerging aquatic insects. Also, to reduce the number of dead amphibian metamorphs and emerging aquatic insects in enclosures or emergence traps as much as possible, I collected the metamorphs on days 51, 63, 65, 72, 74, 84, 89, 94, 100, and 147. Specifically, I collected salamanders from the enclosures when their tail fin shrunk and external gills were absorbed almost completely (stage 68; Iwasawa & Yamashita, 1991). For the froglets, I collected them from the enclosures when their tail was completely absorbed (Gosner stage 46; Gosner, 1960). For emerging aquatic insects, I collected them in the emergence traps placed on the top of enclosures (Figure 2a) and their exuviae floated on the enclosures’ water surface. All aquatic insects and emerging insects were identified to order level (see Appendix B). I terminated the survey on day 147 (16 November) because of the logistical constraints about accessing the field pond (snow cover). Those count data were used to examine how cannibalism among salamander larvae affects the abundance of amphibians and aquatic insects. Moreover, on day 34, I also scanned the ventral aspect of surviving salamander larvae using a scanner (Canoscan 9000F, Canon, Japan). The scanned images were used to measure the body length and gape width of all surviving salamanders. The trait measurements were conducted using Image J (National Institute of Health, USA).

### 2.4 Statistical analysis

All statistical analyses described below were conducted using R (version 3.6.1, R Development Core Team, 2019) within the R studio interface (version 1.2.5001, R studio team, 2019).

#### 2.4.1 Survivorship and morphological traits of salamander larvae

On day 34, I visually confirmed that most enclosures only in the Cannibalism treatment had giant salamander larvae. To statistically examine this pattern, I compared gape width and body length (log‐transformed) of salamander larva with the largest body length in each tank on day 34 using a one‐way analysis of variance. Focusing on a single individual in each enclosure is reasonable because cannibalistic giants emerge in a very low proportion of a population (Kishida, 2011). Also, I examined the effects of cannibalism treatment on survivorship of salamander larvae until day 34 using a generalized linear model (GLM) with a quasi‐binomial error distribution (logit‐link) to correct overdispersion (glm function in stats package).

#### 2.4.2 Prey demographic variables

I examined the effects of treatment on survivorship of frog tadpoles during the experiment using GLM with a quasi‐binomial error distribution (logit‐link) followed by Tukey's HSD post‐hoc test. Since all surviving frog tadpoles had metamorphosed by day 51 (Figure E1 in Appendix E), I used the total number of frog metamorphs during the experiment to calculate the survivorship of the frog tadpoles. On the other hand, calculating the survivorship of aquatic insects was impracticable because of the lack of data regarding immigration to and emigration fromenclosures. Instead of survivorship, I compared the number of insects remaining in the enclosures and the total number of emerging aquatic insects among treatments. Therefore, in this experiment, I cannot exclude the possibility that differences in the focal prey demographic variables among treatments were caused by differences in immigration and emigration rates among treatments. However, it is expected that any difference in the focal demographic variables of aquatic insects among treatments was mainly caused by survivorship differences among treatments. This is because environmental water containing predator chemical cues, which are thought to be important in determining prey behaviors, especially in aquatic ecosystems (Blaustein et al., 2004; Kats & Dill, 1998; Vonesh et al., 2009), was shared across the experimental enclosure in the field pond (Figure 2a). To compare the number of aquatic insects remaining in the enclosures among treatments, I performed generalized linear mixed models (GLMM) with quasi‐Poisson error distribution (log‐link) using the glmmPQL function in the MASS package. In the models, I considered treatment as an explanatory variable and census timing as a covariate. Interaction between treatment and the census timing was included as an additional explanatory variable in the models. Enclosure ID was included as a random factor to give a repeated measured design. Since I could not find any aquatic insects before day 34 (Figure 4a–c), I used data collected on days 34, 44, 57, 109, and 145 for the analyses. When a significant treatment effect was detected, but the interaction between temperature and census timing was not significant, I removed the interaction from the model. I then performed post‐hoc mean comparisons using Tukey's HSD to identify treatments that significantly differ from each other. When the interaction effect was significant, I performed GLM with quasi‐Poisson error distribution (log‐link) followed by Tukey's HSD post‐hoc test for each census timing separately with treatment as an explanatory variable. To compare the total number of the emerging aquatic insects of each taxon, I performed GLM with a quasi‐Poisson error distribution (log‐link) followed by Tukey's HSD post‐hoc test. I did not perform the GLM on Hemiptera since there was no emergence of Hemiptera (Table B2 in Appendix B).

## 3 RESULTS

There were no significant effects of salamander hatch timing difference (No‐cannibalism ‐early vs. ‐late) on any of the focal demographic parameters (see Appendix C). Hence, the data from the two No‐cannibalism treatments were pooled before conducting the following statistical analyses (hereafter, No‐cannibalism treatment).

### 3.1 Survivorship and morphological traits of salamander larvae

Survivorship of salamander larvae until day 34 in the Cannibalism treatment (36.4 ± 15.6% [mean ± *SD*]) was less than half of that in the No‐cannibalism treatment (95.8 ± 3.8%) (GLM with a quasi‐binomial error distribution [logit‐link], $\chi_{1}^{2}$ = 201.69, *p* < .0001 [Figure D2 in Appendix D]). Body length of individuals with the largest body length in the Cannibalism treatment (33.1 ± 3.2 mm) was 1.7 times larger than that in the No‐cannibalism treatment (19.1 ± 1.3 mm) (*t*‐test, *t*_28_ = 17.35, *p* < .0001) (Figure 1b). Gape width of the largest salamander in the Cannibalism treatment (9.7 ± 1.3 mm) was 1.7 times larger than that in the No‐cannibalism treatment (5.7 ± 0.6 mm) (*t*_28_ = 11.70, *p* < .0001) (Figure 1b).

### 3.2 Prey demographic variables

#### 3.2.1 Survivorship of frog tadpoles

**FIGURE 3:**
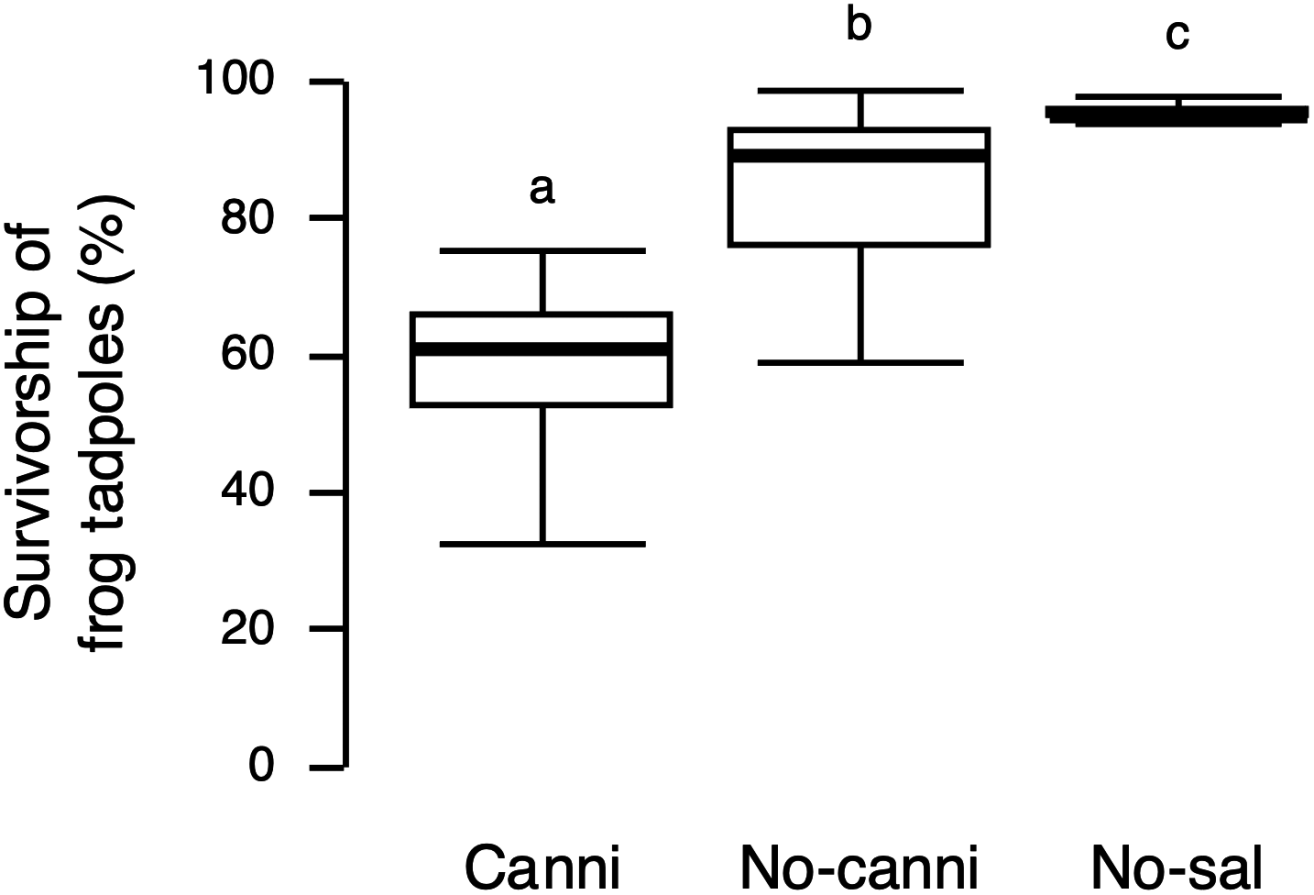
Survivorship of the frog tadpoles during the experimental period. The thick horizontal bars represent the median, the box contains 50% of the data, and the whiskers indicate the range. Treatments not sharing the same lowercase letter were significantly different from each other (Tukey's HSD: *p* < .05). See Figure 2 for abbreviations

Survivorship of frog tadpoles during the experiment significantly differed among treatments (GLM with quasi‐binomial error distribution [logit‐link], $\chi_{2}^{2}$ = 62.50, *p* < .0001) (Figure 3). Post‐hoc pairwise comparisons using Tukey's HSD found that survivorship of frog tadpoles in the Cannibalism treatment (58.8 ± 12.0% [mean ± *SD*]) was 31% and 38% lower than the No‐cannibalism (84.9 ± 11.6%; *z*‐ratio = −5.80, *p* < .0001) and the No‐salamander (95.4 ± 1.3%; *z*‐ratio = −4.95, *p* < .0001) treatments, respectively. Survivorship of frog tadpoles in the No‐cannibalism treatment was 11% lower than the No‐salamander treatment (*z*‐ratio = −2.36, *p* = .048). These results indicate that cannibalism among salamander larvae increased predation on frog tadpoles.

#### 3.2.2 Number of aquatic insects remaining in the enclosures and total number of emerging aquatic insects

**FIGURE 4:**
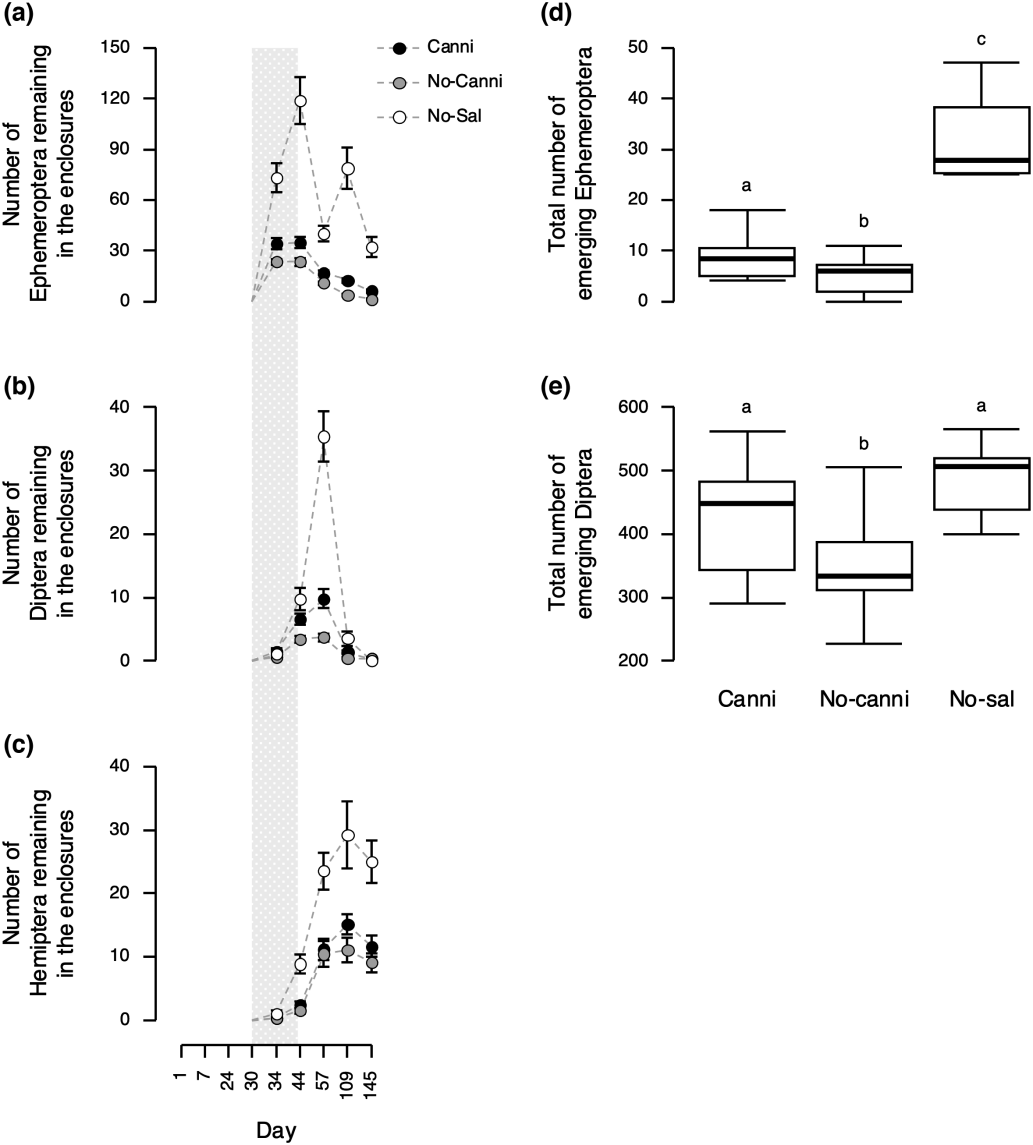
The numbers of (a) Ephemeroptera, (b) Diptera, and (c) Hemiptera remaining in the enclosures over the experimental period. Error bars denote standard error. The shaded area in the figures represents the period when cannibals, non‐cannibals, frog tadpoles, and aquatic insects coexisted within enclosures. The total number of (d) emerging Ephemeroptera and (e) emerging Diptera was collected during the experiment. The thick horizontal bars represent the median, the box contains 50% of the data, and the whiskers indicate the range. Treatments not sharing the same lowercase letter were significantly different from each other (Tukey's HSD: *p* < .05). See Figure 2 for abbreviations

For all aquatic insects (Ephemeroptera, Diptera, and Hemiptera), the number of individuals remaining in the enclosures was significantly affected by both treatment and census timing (*p* < .0001) (GLMM with quasi‐Poisson error distribution [log link]) (Figure 4a–c and Table 1). There was no significant effect of an interaction between treatment and census timing (*p* > .91) except for Ephemeroptera ($\chi_{2}^{2}$ = 50.61, *p* < .0001) (Table 1). Thus, except for Ephemeroptera, I performed GLMM without considering the interaction effects, and then performed post‐hoc pairwise comparisons using Tukey's HSD to identify which treatments differed from each other. The post‐hoc pairwise comparisons found that Diptera (*t*‐ratio = −7.48, *p* < .0001) and Hemiptera (*t*‐ratio = −5.55, *p* < .0001) remaining in the enclosures in the No‐cannibalism treatment were significantly lower than the No‐salamander treatment. Similarly, Diptera (*t*‐ratio = −4.87, *p* = .0001) and Hemiptera (*t*‐ratio = −4.29, *p* = .0004) remaining in the enclosures in the Cannibalism treatment were significantly lower than the No‐salamander treatment. Importantly, the prey demographic variables in the No‐cannibalism treatment were generally lower than the Cannibalism treatment although the difference was significant only for Diptera (*t*‐ratio = 3.51, *p* = .0037) and not for Hemiptera (*t*‐ratio = 1.42, *p* = .34) (Figure 4b,c). Regarding the number of Ephemeroptera remaining in the enclosures, comparisons among treatments on each census timing found that the numbers in both Cannibalism and No‐cannibalism treatments were significantly lower than the No‐salamander treatments across the period during which Ephemeroptera larvae were observed in the enclosures (*p* < .0001 [Table F1 in Appendix F]. Importantly, the numbers of Ephemeroptera in the No‐cannibalism treatment were significantly lower than those in the Cannibalism treatment across the period (*p* < 0.020 [Table F1 in Appendix F]) (Figure 4a).

**TABLE 1:** Summary of results of analyses examining the effects of treatment, census timing, and interaction between them on aquatic insects remaining in the enclosures using generalized linear mixed models (GLMM) with quasi‐Poisson error distribution (log‐link)

Dependent variables	Explanatory variables	*χ^2^*	*df*	*p*
Ephemeroptera remaining in the enclosures	Treatment	233.87	2	<.0001
	Census timing	130.24	1	<.0001
	Treatment*census timing	50.61	2	<.0001
Diptera remaining in the enclosures	Treatment	61.98	2	<.0001
	Census timing	29.99	1	<.0001
	Treatment*census timing	0.068	2	.97
Hemiptera remaining in the enclosures	Treatment	33.27	2	<.0001
	Census timing	67.81	1	<.0001
	Treatment*census timing	0.19	2	.91

Enclosure ID was included as a random factor to give a repeated measured design.

Total number of emerging aquatic insects over the experimental period significantly differed among treatments (GLM with quasi‐Poisson error distribution [log‐link]); Ephemeroptera ($\chi_{2}^{2}$ = 118.51, *p* < .0001), and Diptera ($\chi_{2}^{2}$ = 14.41, *p* = .00074) (Figure 4d,e). Post‐hoc pairwise comparisons found differences among treatments which are consistent with the patterns observed in the former analyses. Total number of emerging Ephemeroptera in the No‐cannibalism treatment (5.2 ± 3.0 [mean ± *SD*]) was 38% and 84% lower than the Cannibalism (8.4 ± 4.0; *z*‐ratio = 2.37, *p* = .047) and No‐salamander (32.3 ± 9.4; *z*‐ratio = −9.94, *p* < .0001) treatments, respectively. Total number of Ephemeroptera metamorphs in the Cannibalism treatment was also 74% lower than the No‐salamander treatment (*z*‐ratio = −8.24, *p* < .0001). Similarly, total number of emerging Diptera in the No‐cannibalism treatment (352.2 ± 74.9 [mean ± *SD*]) was 17% and 28% lower than the Cannibalism (424.6 ± 84.8; *z*‐ratio = 2.58, *p* = .027) and No‐salamander (486.0 ± 64.6; *z*‐ratio = −3.62, *p* = .0009) treatments, respectively. Total number of emerging Diptera in the Cannibalism treatment was 13% lower than the No‐salamander treatment while the difference between treatments was not significant (*z*‐ratio = −1.53, *p* = .28). The differences among treatments consistently observed in aquatic insects remaining in the enclosures and total number of emerging insects suggest that cannibalism among salamanders reduced predation on aquatic insects.

## 4 DISCUSSION

While cannibalism among predators can both reduce and increase predation on heterospecific prey (Persson et al., 2003; Rudolf, 2006, 2012; Takatsu & Kishida, 2015), we know less about the factors that lead to these contrasting outcomes. Here, I tested the hypothesis that cannibalism among predators can reduce predation on small heterospecific prey but increase predation on large heterospecific prey using *H*. *retardatus* salamander larvae and their associated prey community. In the focal prey community, aquatic insects were small prey for salamander larvae, and frog tadpoles were large prey (Figure 1). Consistent with my hypothesis, in the field‐enclosure experiment, while the presence of salamanders reduced the abundance of the small and large prey, the numbers of the aquatic insects remaining in the enclosures and emerging aquatic insects were generally lowest when salamanders did not cannibalize, but survivorship of the frog tadpoles was lowest when salamander cannibalized (Figures 3 and 4). This study provides empirical evidence that the effects of predator cannibalism on prey depend on the prey body size.

There is, however, a possibility that the differences in the direction of predator cannibalism effects among prey items could be solely explained by prey phenology rather than prey body size. In this experiment, the assignment of frog tadpoles (day 1 [23 June]) into the enclosures was about 1 month earlier than the beginning of the natural recruitment of larval aquatic insects (day 30 [22 July]) (Figure 4a–c). Such a difference in recruitment timing is often observed in wild ponds (personal observation). Importantly, cannibals, which are the key individuals causing an increase in predation on heterospecific prey, metamorphose earlier than non‐cannibals partly because of additional energy gain from cannibalism (Sniegula et al., 2017, 2019; Takatsu & Kishida, 2015). Indeed, the timing of the first salamander metamorphosis in the Cannibalism treatment (46.0 ± 3.3 days [mean ± *SD*]) was about a month earlier than the No‐cannibalism treatment (72.3 ± 3.7 days) (see Figure D1b in Appendix D). Therefore, aquatic insects had suffered predation by the cannibals over a far shorter period than frog tadpoles. This can enable cannibalism among salamanders to reduce the predation on the aquatic insects but increase the predation on the frog tadpoles even without consideration of the prey body size. Nevertheless, when focusing only on the prey demographic variables observed during the time period when the cannibals, non‐cannibals, frog tadpoles, and aquatic insects coexisted (i.e., the shaded area in Figure 4a–c), I obtained qualitatively similar results to what was described above. For example, the number of Ephemeroptera remaining in the enclosures on day 34 in the No‐cannibalism treatment (23.4 ± 7.1 [mean ± *SD*]) was 31% and 68% lower than Cannibalism (34.1 ± 13.2; *z*‐ratio = 2.75, *p* = .017) and No‐salamander (73.2 ± 21.0; *z*‐ratio = −8.20, *p* < .0001) (GLM with quasi‐Poisson error distribution [log‐link]), respectively (Figure 4a). These results suggest that cannibalism among salamanders reduced predation on aquatic insects. On the other hand, mortality (number of dead individuals) of frog tadpoles between day 30 and 34 in the Cannibalism treatment (7.1 ± 3.5 [mean ± *SD*]) was 5.7 times and 4.2 times greater than No‐cannibalism (1.3 ± 1.2; *z*‐ratio = 6.16, *p* < .0001) and No‐salamander (1.7 ± 1.0; *z*‐ratio = 3.79, *p* = .0004) (GLM with quasi‐Poisson error distribution [log‐link]), respectively (Figure E1c in Appendix E). These results indicate that cannibalism among salamanders increased predation on the frog tadpoles during that time period. These results further support the conclusion that the direction of predator cannibalism effects on the strength of predation can differ depending on prey body size, although this study does not exclude the importance of the prey phenology in determining the direction of predator cannibalism effects.

There are two possible mechanisms causing the prey body size dependency in the effects of salamander cannibalism. Firstly, aquatic insects were within the consumable size range of both non‐cannibals and cannibals, although frog tadpoles were too large for non‐cannibals to consume (Figure 1b). As a result, the reductions in non‐cannibals’ density and foraging activity due to cannibalism were less likely to affect the predation of the frog tadpoles. Therefore, the strength of the predation on the frog tadpoles was solely determined by the effects of the improvement of cannibals’ foraging ability, as shown in the previous studies (Takatsu & Kishida, 2015). At the same time, the predation on the aquatic insects was likely to be affected by reductions in non‐cannibals’ density and foraging activity and improvement of cannibals’ foraging ability. However, possibly because only a few individuals within a population can cannibalize and become giant cannibals (Kishida, 2011), the effects of improvement of the foraging ability of the few cannibals cannot overwhelm the effects of reductions in non‐cannibals’ density and foraging activity. Indeed, there were less than five salamanders whose gape size was larger than the body width of frog tadpoles, while cannibalism reduced the population by less than half (Figure D2‐3 in Appendix D). Secondly, there is a possibility that the diet broadening of cannibals reduced the frequency of the aquatic insects being consumed by the cannibals. This can make the reductions in non‐cannibals’ density and foraging activity more significant in determining the net effects of predator cannibalism on the strength of predation on aquatic insects. To test the possibility, I examined the relationship between the mortality of frog tadpoles and the abundance of the aquatic insects during the period when frog tadpoles and aquatic insects coexisted (i.e., the shaded area in Figure 4a–c). In the analysis, I used only data from the Cannibalism treatment since the frog tadpoles in the No‐cannibalism treatment were rarely consumed by salamanders (Figure 3). If consumption of frog tadpoles reduced the frequency of aquatic insects being consumed by salamander larvae, there should be a positive relationship between the mortality of frog tadpoles and the abundance of aquatic insects. However, there was no significant relationship between the mortality of frog tadpoles between days 30 and 34 and the number of Ephemeroptera remaining in the enclosures on day 34 (GLM with quasi‐Poisson error distribution, $\chi_{1}^{2}$ = 0.012, *p* = .91) (Figure G1 in Appendix G). Therefore, it is expected that the relative size relationships between salamander larvae and their prey were more important than the diet broadening of cannibals in explaining the contrasting effects of salamander cannibalism on predation.

Considering that predators often broaden their diet and or shift their diet from small to large prey with their growth (Werner & Gilliam, 1984), paying attention to the size composition of prey communities is likely to be essential when predicting community‐level consequences of predator cannibalism. For example, suppose that a focal prey community is dominated by large prey species (e.g., frog tadpole in this study system). Then, cannibalism among predators can shift the prey community toward dominance by small prey species by increasing predation on large prey but reducing predation on small prey simultaneously. Importantly, as well as species identity, the body size is considered as an important factor in explaining variation in individuals’ ecological roles since body size is correlated with, for instance, trophic position, consumption rate, and nutrient excretion rate (Romanuk et al., 2011; Vanni et al., 2002; Werner & Gilliam, 1984; Woodward et al., 2005). Partly due to this, large species often play a disproportionately large role in determining ecosystem functions, such as primary production (Séguin et al., 2014). All this considered, the shift in the prey community toward dominance by small prey species due to predator cannibalism could have significant impacts on ecosystem function. To further our understanding of community‐level consequences of predator cannibalism, examining how predator cannibalism effects change along with the size composition of prey communities and how the effects of predator cannibalism on prey community composition are eventually reflected in ecosystem function will be fruitful next steps.

While our knowledge of the ecological consequences of predator cannibalism has gradually accumulated (Crumrine, 2010a; Persson et al., 2003; Rudolf, 2006, 2012; Takatsu & Kishida, 2015, 2020; Takatsu et al., 2017), community‐level consequences of predator cannibalism are still unclear. Here, I showed that cannibalism among predators can reduce predation on small prey species but increase predation on large prey species simultaneously. Previous studies showed that the occurrence and strength of cannibalism among predators are highly dependent on surrounding environmental conditions, including the presence and absence of top predators, ambient temperature, and changes in water level (Crumrine, 2010b; Gillespie et al., 2020; Kishida, 2011; Sniegula et al., 2019). All this considered, cannibalism among predators could play a pivotal role in establishing links between biotic and abiotic factors and predator's effects on the prey community and resultant cascading effects on lower trophic levels. Investigating the context‐dependency of cannibalism among predators further and how such context‐dependent cannibalism among predators affects the nature and strength of top‐down effects might be essential next steps in furthering our understanding of community‐ and ecosystem‐level consequences of predator cannibalism.

## AUTHOR CONTRIBUTIONS

**Kunio Takatsu:** Conceptualization (equal); Data curation (equal); Formal analysis (equal); Funding acquisition (equal); Investigation (equal); Methodology (equal); Project administration (equal); Writing – original draft (equal); Writing – review & editing (equal).

## Supporting information

Supplementary Material

## Data Availability

Data available from the Dryad Digital Repository https://doi.org/10.5061/dryad.8cz8w9gt5 (Takatsu 2022).
