# Supplementary material for: Predator cannibalism can shift prey community composition toward dominance by small prey species
Source: Ecol Evol. 2022 May 7;12(5):e8894. doi: 10.1002/ece3.8894 (PMC9077740; doi:10.1002/ece3.8894)
Supplement: Supplementary file 1 — Supplementary Material [file ECE3-12-e8894-s001.docx]

**APPENDIX A:** Methods of animal collection and husbandry

I collected 30 egg clusters of *Hynobius retardatus* salamander and ten egg masses of *Rana pirica* frog from several ponds in Teshio Experimental Forest of Hokkaido University late-May 2013. In general, I kept the collected salamander egg cluster and frog masses following the methods used in the previous studies (Takatsu and Kishida 2015 [Appendix A], 2020 [Appendix A], Takatsu et al. 2017 [Appendix S1]).

Each of the 30 salamander egg clusters was reared separately in fine mesh nets. The nets were placed in a 4 L semi-transparent polypropylene tank (33.4 cm × 20 cm × 10 cm high; 5 nets per tank) filled with 2 L of aged tap water. To control the hatch timing, tanks were then placed in a refrigerator maintained at 3 ℃ under natural day/night (about 14h/10h) conditions. One week before the start of the field experiment, when almost all of the embryos had reached developmental stage 38-39 (Iwasawa and Yamashita 1991), I carried out manipulation to obtain early- and late- salamander hatchlings to establish Cannibalism and No-cannibalism treatments. The methodological details to obtain early- and late- salamander hatchlings are the same as those shown in Appendix A in Takatsu and Kishida (2015).

Each of the 10 frog egg masses was kept in a separate 22 L semi-transparent polypropylene tank (43.6 cm × 28.4 cm × 14.1 cm high) filled with 5 L of aged tap water. Tanks were placed in a controlled environment room and maintained at 17 ℃ with a natural day/night (about 14/10h) regime. The egg had started to hatch in early June. After the frog tadpoles had hatched, I put eight pieces of rabbit chow (dry weight: 1.6 g) into each tank as a food source and exchange the water every two days until the start of the experiment (i.e., June 23 [day 1 of the experiment]).

**References**

Iwasawa, H. and K. Yamashita. 1991. Normal stages of development of a hynobiid salamander, *Hynobius nigrescens* Stejneger. – Jap J. Herpet. 14: 39-62.

Takatsu, K. and Kishida, O. 2015. Predator cannibalism can intensify negative impacts on heterospecific prey. – Ecology 96: 1887-1898.

Takatsu, K. and Kishida, O. 2020. Enhanced recruitment of larger predators in the presence of large prey. – J. Anim. Ecol. 89: 1615-1627.

Takatsu, K. et al. 2017. Giant cannibals drive selection for inducible defense in heterospecific prey. – Biol. J. Linne. Soc. 120: 675-684.

**APPENDIX B**: Orders of aquatic insects collected over the experimental period

**Table B1.** Orders of aquatic insects remaining in the enclosure observed over the experimental period. The numbers represent the total numbers of aquatic insects remaining in the enclosures across all replicates (i.e., 36 enclosures) at each census timing.

| 145 | 300 | 10 | 458 | 1 | 15 |
| --- | --- | --- | --- | --- | --- |
| 109 | 703 | 47 | 562 | 1 | 15 |
| 57 | 648 | 408 | 464 | 0 | 10 |
| 44 | 1574 | 203 | 111 | 2 | 11 |
| 34 | 1291 | 34 | 15 | 1 | 1 |
| 30 | 0 | 0 | 0 | 0 | 0 |
| 24 | 0 | 0 | 0 | 0 | 0 |
| 7 | 0 | 0 | 0 | 0 | 0 |
| 1 | 0 | 0 | 0 | 0 | 0 |
| Census timing (day) | Epemeroptera | Diptera | Hemiptera | Coleoptera | Odonata |

**Table B2.** Orders of aquatic insect emergence over the experimental period. The numbers represent the total numbers of aquatic insect emergence across all replicates (i.e., 36 enclosures) at each census timing.

| 147 | 0 | 0 |
| --- | --- | --- |
| 145 | 0 | 0 |
| 109 | 0 | 0 |
| 100 | 0 | 725 |
| 94 | 0 | 2515 |
| 89 | 0 | 1871 |
| 84 | 3 | 3058 |
| 74 | 12 | 1806 |
| 72 | 35 | 1658 |
| 65 | 149 | 2231 |
| 63 | 65 | 472 |
| 57 | 130 | 159 |
| 51 | 0 | 0 |
| 44 | 0 | 0 |
| 34 | 0 | 0 |
| Census timing (day) | Epemeroptera | Diptera |

**APPENDIX C:** Summary of statistical analyses comparing No-cannibalism -early and -late treatments.

I initially established No-cannibalism -early and -late treatments. Consistent with results from the previous studies (Takatsu and Kishida 2015, 2020, Takatsu et al. 2017), the difference in hatch timing (1 week) between the treatments had no effects on any of the focal salamander demographic variables as described in the following tables. Moreover, the differences in the hatch timing and interaction between hatch timing and census timing also had no effects on any of the focal prey demographic variables. Therefore, I pooled the data of the two No-cannibalism treatments.

**Table C1.** Result of the generalized linear model (GLM) with a quasi-binomial error distribution (logit-link) comparing survival of salamander larvae until day 34 between the two No-cannibalism treatments.

| **Dependent variables** | ***χ^2^*** | **d.f.** | ***P*** |
| --- | --- | --- | --- |
| Survival until day 34 | 0.19 | 1 | 0.66 |

**Table C2.** Results of the *t*-test comparing gape width of- and body length of- the salamander larva with the largest body length in each tank at day 34 between the two No-cannibalism treatments.

| **Dependent variables** | ***t*_14_** | ***P*** |
| --- | --- | --- |
| Gape width at day 34 | 1.59 | 0.13 |
| Body length at day 34 | 1.85 | 0.085 |

**Table C3.** Summary of results of analyses examining the effects of treatment, census timing, and interaction between them on number of aquatic insects (Ephemeroptera, Diptera, and Hemiptera) remaining in the enclosures using generalized linear mixed models (GLMM) with quasi-Poisson error distribution (log-link) to correct overdispersion. Enclosure ID was included as a random factor to give a repeated measured design.

| **Dependent variables** | **Explanatory variables** | *χ^2^* | **d.f.** | ***P*** |
| --- | --- | --- | --- | --- |
| Ephemeroptera  remaining in the  enclosures | Treatment | 3.47 | 1 | 0.063 |
|  | Census timing | 155.51 | 1 | < 0.0001 |
|  | Treatment*Census timing | 2.68 | 1 | 0.10 |
| Diptera remaining  in the enclosures | Treatment | 0.036 | 1 | 0.85 |
|  | Census timing | 16.02 | 1 | < 0.0001 |
|  | Treatment*Census timing | 0.0008 | 1 | 0.98 |
| Hemiptera remaining in  the enclosures | Treatment | 2.85 | 1 | 0.091 |
|  | Census timing | 23.89 | 1 | < 0.0001 |
|  | Treatment*Census timing | 0.070 | 1 | 0.79 |

**Table C4.** Result of the generalized linear model (GLM) with a quasi-binomial error distribution (logit-link) comparing survival of frog tadpoles during the experiment between the two No-cannibalism treatments.

| **Dependent variables** | ***χ^2^*** | **d.f.** | ***P*** |
| --- | --- | --- | --- |
| Survivorship | 3.16 | 1 | 0.076 |

**Table C4.** Result of the generalized linear model (GLM) with a quasi-Poisson error distribution (log-link) comparing the total number of emerging Ephemeroptera and Diptera collected during the experimental period between the two No-cannibalism treatments.

| **Dependent variables** | ***χ^2^*** | **d.f.** | ***P*** |
| --- | --- | --- | --- |
| Ephemeroptera emergence | 3.21 | 1 | 0.073 |
| Diptera emergence | 0.0030 | 1 | 0.96 |

**References**

Takatsu, K. and Kishida, O. 2015. Predator cannibalism can intensify negative impacts on heterospecific prey. – Ecology 96: 1887-1898.

Takatsu, K. and Kishida, O. 2020. Enhanced recruitment of larger predators in the presence of large prey. – J. Anim. Ecol. 89: 1615-1627.

Takatsu, K. et al. 2017. Giant cannibals drive selection for inducible defense in heterospecific prey. – Biol. J. Linne. Soc. 120: 675-684.

**APPENDIX D:** Demographic variables of salamanders over the experimental period.

**Figure D1.** (a) Number of salamander larvae remaining in the enclosures, (b) number of salamander metamorphs, and (c) mortality (number of dead individuals) of salamander larvae. Error bars denote standard error. The shaded areas in the figures represent a period during which cannibals, non-cannibals, frog tadpoles, and aquatic insects coexisted.

**Figure D2.** Survivorship of salamander larvae until day 34. Error bars denote standard error. Canni and No-canni are abbreviations of Cannibalism and No-cannibalism treatments, respectively.

**
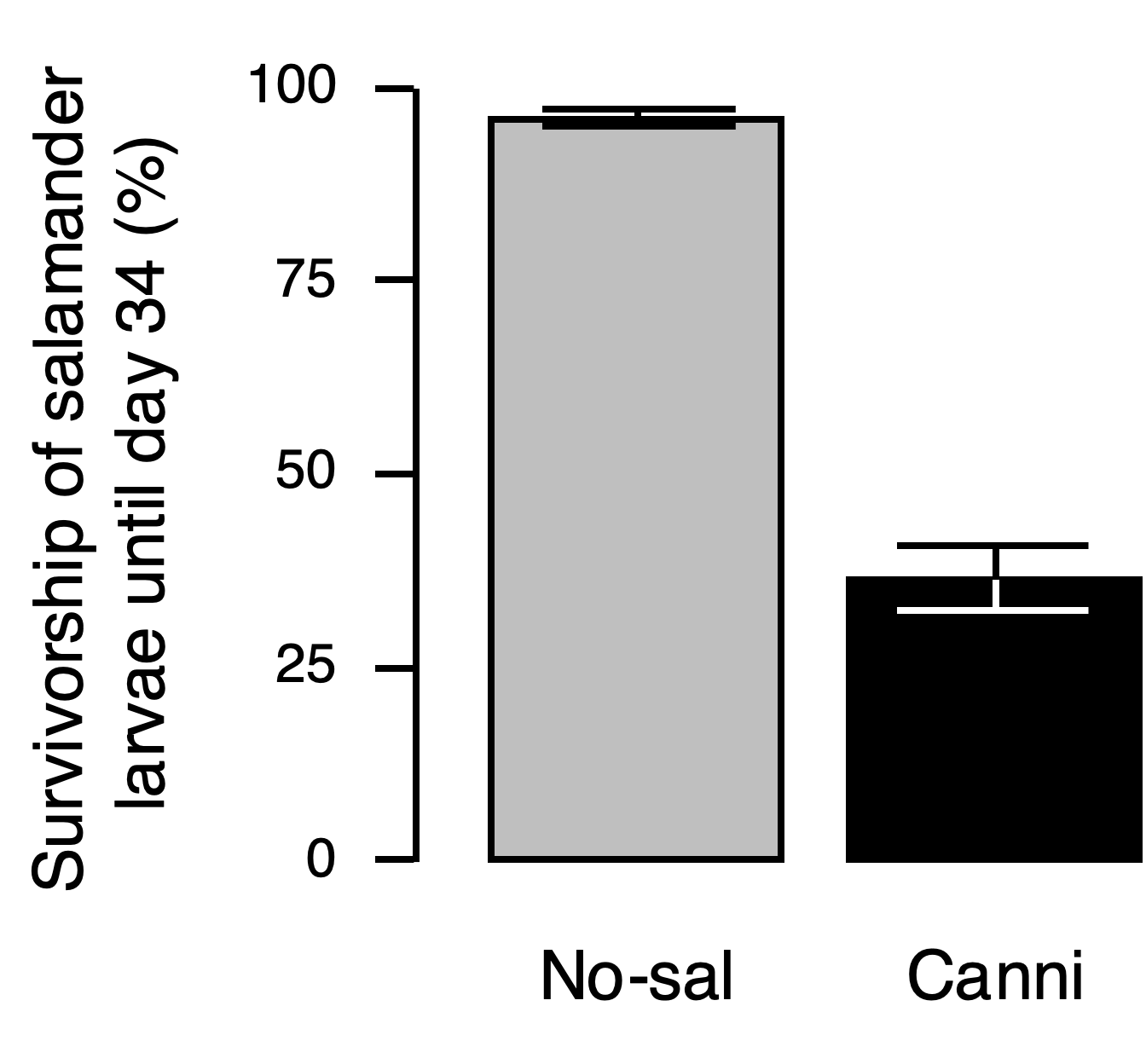
 Figure D3.** Relationship between size (body length) rank and gape size of salamanders at day 34 and body width of frog tadpoles at day 1 of the experiment. The thick horizontal bars represent median, the box contains 50% of the data, and the whiskers indicate the range.

**
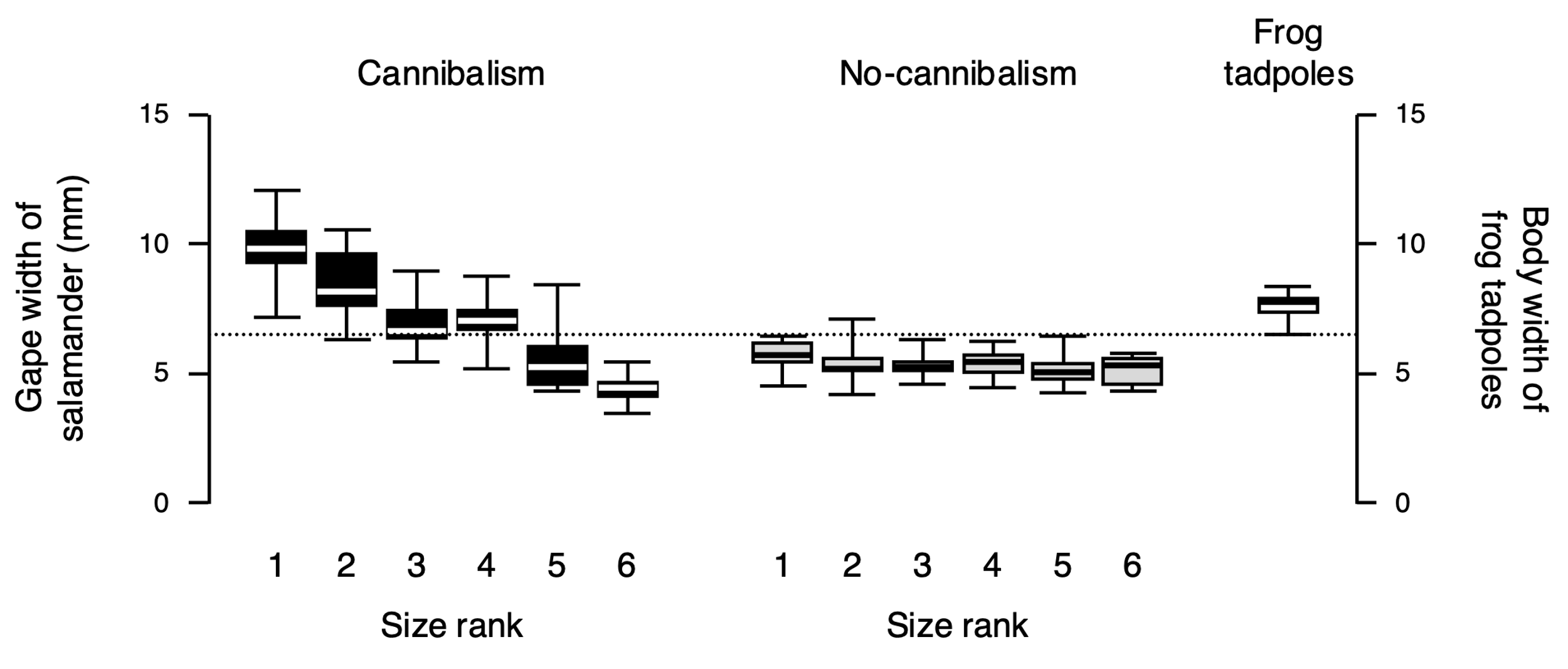
**

**APPENDIX E:** Demographic variables of prey over the experimental period.

**Figure E1.** (a) Number of frog tadpoles remaining in the enclosures, (b) number of frog metamorphs, and (c) mortality (number of dead individuals) of frog tadpoles. Error bars denote standard error. The shaded areas in the figures represent a period during which cannibals, non-cannibals, frog tadpoles, and aquatic insects coexisted.

**Figure E2.** Number of (a) emerging Ephemeroptera and (b) emerging Diptera over the experimental period. Error bars denote standard error. The shaded areas in the figures represent a period during which cannibals, non-cannibals, frog tadpoles, and aquatic insects coexisted.

**APPENDIX F:** Summary of post-hoc pairwise comparisons of Ephemeroptera remaining in the enclosures at each census timing

**Table F1.** Summary of the results of post-hoc pairwise comparisons of Ephemeroptera remaining in the enclosures at each census timing. Canni, No-canni, and No-sal are abbreviations of Cannibalism, No-cannibalism, and No-salamander treatments, respectively.

| **Census timing** | **Pairwise comparisons** | *z-*ratio | ***P*** |
| --- | --- | --- | --- |
| Day 34 | Canni vs. No-canni | 2.75 | .017 |
|  | Canni vs. No-sal | -5.85 | <.0001 |
|  | No-canni vs. No-sal | -8.20 | <.0001 |
| Day 44 | Canni vs. No-canni | 2.68 | .020 |
|  | Canni vs. No-sal | -9.59 | <.0001 |
|  | No-canni vs. No-sal | -11.70 | <.0001 |
| Day 57 | Canni vs. No-canni | 3.21 | .0038 |
|  | Canni vs. No-sal | -7.45 | <.0001 |
|  | No-canni vs. No-sal | -10.14 | <.0001 |
| Day 109 | Canni vs. No-canni | 4.32 | <.0001 |
|  | Canni vs. No-sal | -10.73 | <.0001 |
|  | No-canni vs. No-sal | -11.39 | <.0001 |
| Day 145 | Canni vs. No-canni | 4.06 | .0001 |
|  | Canni vs. No-sal | -8.38 | <.0001 |
|  | No-canni vs. No-sal | -9.17 | <.0001 |

**APPENDIX G:** Relationship between mortality of frog tadpoles and abundance of aquatic insects.

**Figure G1.** Relationship between mortality (number of dead individuals) of frog tadpoles between day 30 to 34 and the number of Ephemeroptera reamining in the enclosures at day 34 in the Cannibalism treatment.
